# Do state laws reduce uptake of Medicaid/CHIP by U.S. citizen children in immigrant families: evaluating evidence for a chilling effect

**DOI:** 10.1186/s12939-022-01651-2

**Published:** 2022-04-12

**Authors:** Sylvia E. Twersky

**Affiliations:** Department of Public Health, The College of New Jersey School of Nursing, Health, and Exercise Science, Ewing, NJ USA

**Keywords:** Medicaid, CHIP, Immigrant, State law, Chilling effect, Health insurance

## Abstract

**Background:**

Restrictive state laws aimed at immigrants can have unintended consequences for health insurance coverage of United States citizens in immigrant families due to both actual barriers created by the laws and perceived barriers among immigrants. Increasing numbers of children in the U.S. are part of immigrant families, and these children are more likely to be living in poverty than their counterparts in native families. Immigrant restrictive policies could lead to reduced access to Medicaid and CHIP even for citizen children in immigrant families.

**Methods:**

Using data from the Current Population Survey's (CPS) March Supplement, linear probability models with difference-in-differences (DD) estimates compare probability of enrollment in Medicaid/CHIP among low-income U.S. citizen children in immigrant families and low-income children in native families and U.S. citizen children in immigrant families in states that did not adopt restrictive legislation, in order to estimate the impact of restrictive state laws aimed at immigrants. An additional model explores the effect of mother’s citizenship on enrollment among all immigrant families in states with and without restrictive legislation.

**Results:**

Results suggest a significant chilling effect where the magnitude of the effect varies according to family demographics and by the types of laws being passed. Immigrant restrictive social welfare laws being adopted have a strong negative effect on U.S. citizen children in immigrant families’ enrollment in Medicaid/CHIP, a 5.5 percentage point reduction in coverage. Among the subsample of only immigrant families, results point toward a global chilling effect created by an overall restrictive policy environment. All immigrant restrictive related laws, including those aimed at education, job restriction, identification access, and social welfare restrictions have a significant and negative impact on access to public insurance for U.S. citizen children with non-citizen mothers.

**Conclusions:**

This research shows that the unintended consequence of restrictive state legislation aimed at immigrants is the reduction in access to Medicaid and CHIP by low-income U.S. citizen children living in immigrant families. Reduced access to health insurance can increase unmet medical needs for an already vulnerable group.

**Supplementary Information:**

The online version contains supplementary material available at 10.1186/s12939-022-01651-2.

## Introduction

This study examines the impact of state laws governing education, regulation, and social welfare on the decision by eligible low-income immigrant families to enroll their U.S. citizen children in Medicaid and the Children's Health Insurance Program (CHIP), using variation in the presence of restrictive state laws over time and across states to identify their impact. Health insurance is one critical factor that contributes to health outcomes. Research regarding children and Medicaid has shown consistently that Medicaid results in increased access to healthcare and improved health outcomes among low-income children [1–3]. Bronchetti showed that Medicaid/CHIP eligibility expansions specifically increased immigrant children's use of preventive and ambulatory care and decreased emergency care in hospitals, as well as increasing health insurance enrollment among children in immigrant families by 23 percentage points [4]. Health insurance significantly reduces financial barriers to accessing healthcare and therefore leads to increased use of primary care [5–7]. In addition, there is an increasing body of evidence showing long term impacts of Medicaid access on child development into adulthood, including lower rates of chronic disease [8, 9], higher educational attainment [8, 10], and higher wages [11]. Thus, state laws that affect eligible children’s enrollment in this safety net program can have a large impact on health care access and outcomes as well as life course development.

Historically, lawfully present non-citizen immigrants (LPI) were eligible for public insurance until the passage of the Personal Responsibility and Work Opportunity Reconciliation Act (PRWORA) in 1996 which restricted access for LPI immigrants based on their immigration status and years in the U.S. Some few states chose to extend Medicaid to previously eligible LPI immigrants or certain groups of immigrants that were excluded under the 1996 PRWORA using state only funds. Multiple studies have shown that PRWORA created a chilling effect on eligible immigrants’ enrollment [12–14]. The concept of a chilling effect is derived from constitutional law [15] and has been applied to welfare reform under PRWORA, including specifically Medicaid access. In this context it is defined as when eligible immigrants withdraw or refrain from accessing public benefit programs because of confusion over eligibility or fear that it will negatively impact their or their family’s immigration status [16–18]. This may reflect the perception that use of social safety net programs could affect citizenship applications, although the use of Medicaid or CHIP does not count toward a public charge during the study period. A public charge is defined as a person who is considered dependent on the government for cash or long-term care. An immigrant who is found likely to become a public charge may be denied lawful permanent resident status [19]. Under the Trump administration in 2019 the public charge rule was expanded to include Medicaid use, but this was subsequently suspended in March 2021, and outreach to the affected community regarding this change is ongoing [20]. While there is strong historical data supporting the chilling effect of federal legislation on social safety net enrollment, there is little in the way of research looking at state level legislation and its potential to create a chilling effect on enrollment among immigrant families. This study attempts to fill that gap while also providing a magnitude of effect. A study by Kandula, et. al. suggests that state level anti-immigrant laws that are not directly aimed at public insurance access can still create a chilling effect on this access for immigrants [12].

This chilling effect can equally be seen in the coverage of native children with immigrant parents. Native children are always eligible for public insurance if they meet income requirements. Yet, multiple studies show a significant gap in insurance between citizen children with one or more non-citizen parents and children with citizen parents [13, 14, 18]. Kaushal and Kaestner found that PRWORA itself was at least partially responsible for this difference in coverage [14] and Acevedo-Garcia and Stone show considerable state variation in insurance coverage gaps for children with a non-citizen parent [18].

Across all states children in immigrant families are more likely to be uninsured. Multiple studies show higher rates of uninsured children in immigrant families compared to native families, as well as state differentials in access by immigrant families [14, 21–23]. In addition, the makeup of the immigrant family in terms of native vs. non-native parents has been shown to have an effect on children’s insurance access. This effect is differential by state [22, 23]. Medicaid and CHIP enrollment by eligible children varies by state due to differences in state outreach, enrollment, and eligibility practices. This indicates that state political characteristics and the environment toward immigrants may pay a role in the likelihood of U.S. citizen children in immigrant families accessing safety net insurance services. Public insurance is a key safety net program for children in immigrant families. Thus, understanding the mechanisms for the differential in public insurance uptake is critical. Due to a chilling effect, immigrant parents may be less likely to enroll their citizen children in Medicaid/CHIP in states with restrictive immigrant legislation compared to a group that is not affected by those laws.

### Methodology of analysis

The challenge facing this analysis is to identify whether restrictive state laws that target immigrant status have a causal effect on the outcome of interest: enrollment by U.S. citizen children in immigrant families in Medicaid /state CHIP (SCHIP). To identify this effect, the study treats the implementation of such state laws as a natural experiment and uses variation in the timing of adoption and the nature of the laws across states and over time to identify their impact. To do so, the study applies the quasi-experimental difference-in-differences (DD) estimation approach. This approach considers whether over the period encompassing 2000 to 2008, the enactment of restrictive laws had a differential effect on the enrollment in Medicaid/CHIP by a “treatment” group of U.S. citizen children in immigrant families compared to a control group of U.S. citizen children in native families and U.S. citizen children in immigrant families in states that did not adopt restrictive legislation. This analysis period reflects a time after PRWORA but before the passage of the Affordable Care Act (ACA), in order to try and isolate the effect of state laws on public insurance uptake without confounding due to changes in federal regulations.

The DD identification strategy eliminates the influence of any unobserved, time- invariant differences between states that adopted and did not adopt social safety net legislation that may have constrained enrollment by non-native immigrants. This eliminates any time-invariant differences between the states that might be correlated with the adoption of such legislation and the outcome of interest and would yield biased estimates of the impact of the state laws. This will be further controlled for through the use of state fixed effects, which control for unobserved heterogeneity across states and yields estimates of the within-state change in the outcome of interest. Additionally, the DD approach controls for unobserved time-varying differences that are common to both sets of states. There is some concern in recent literature that linear policy DD analysis can also reflect time-varying treatment effects [24]. The concern is addressed here through analysis of the isolated “treatment” of policy exposure for each one year wave (see Regression analysis section on independent variables). In addition restrictive policy adoption is not a de novo policy change, but rather builds on previous policy exposure (see Discussion under state characteristics) such that the weighted average Goodman-Bacon treatment effect [24] of early, late and never adopting may not clearly apply. Instead, previous policy exposure is controlled for through a variable looking at previous state generosity in regard to social safety net programs for immigrants. For a discussion of the state generosity variable please see the State Characteristics subsection. Additionally, to assess the sensitivity of this analysis to potential dynamic treatment effects, a subset analysis looking just at variation among ever “treated” is performed by a DD analysis on immigrant families only.

The main dataset used for this analysis is the March Supplement of the Current Population Survey (CPS) from 2000 to 2008 [25]. The March Supplement to the CPS is the annual socioeconomic supplement to the U.S. government’s monthly labor force survey and is both nationally and state-level representative. The data collected in the March Supplement oversamples minorities and it is regularly used in studies that look specifically at immigrant populations [26]. The information needed to identify immigrants including country of birth, citizenship, and year of entry is part of the CPS dataset, and family data is linked so that it is possible to identify immigrant parents with U.S. citizen children. In March of every year the sample size for the CPS is increased and additional data is collected. For this analysis the key variables include information on income and health insurance, including participation in public insurance. The CPS data was merged with a state law dataset [27] that includes the law data by state and year and state specific characteristics by year. To identify the applicable state laws, a search was conducted through the Lexis Nexus Federal and State Codes, Advanced Legislative Service-50 states, DC, PR, and VI combined. The laws were further characterized as educational rights, employment regulation, and social service use. For full details on the methodology of the law dataset construction and a discussion of the laws please see Supplement 1.

### Defining treatment and control groups

Using the same methodology as a previous analysis looking at Supplemental Nutrition Assistance Program benefits among immigrant families [28], the analysis is restricted to 20 states at or above the U.S. average for percentage of foreign-born population (traditional gateway states) or a state that ranked in the top 10 percent in increase in foreign-born population (new destination states) during the analysis time period. This reflects the idea that states that have a large population of immigrants or states that are experiencing a surge in the immigrant population may seek to address increases in the immigrant population through legislation. States representing criteria one (traditional gateway states) include: Arizona, California, Connecticut, Florida, Illinois, Massachusetts, New Jersey, New York, Rhode Island and Texas. States representing criteria two (new destination states) include: Alabama, Arkansas, Delaware, Georgia, Kentucky, Mississippi, North Carolina, South Carolina and Tennessee. Nevada meets both criteria for inclusion.

The treatment group consists of children in the analysis states where at least one parent is an immigrant to the U.S. Buchmueller, Sasso, and Wong, in their analysis of CHIP coverage of children in immigrant families, showed that identifying children in immigrant households in this way provides the same results as the method used by Borjas of identifying these children by the nativity of the head of household [29, 30]. Children for whom it was not possible to identify the nativity of their parents were excluded from the analysis. Any children that were not U.S. citizens were also excluded from the analysis. The treatment group was thus restricted to children, 18 and under, who are U.S. citizens by naturalization or birth, who are in families with income 200% of the federal poverty level or below for the year under analysis, and who have at least one immigrant parent, regardless of the parents’ citizenship. The reason for restricting the analysis to U.S. citizen children is that it is the only way to be sure of eligibility, since the CPS does not differentiate between legal and undocumented immigrants. In 2006, 86% of children in the U.S. with immigrant parents were U.S. citizens [31]. The control group consists of children, ages 18 and under, in the same 20 states with two native parents as well as U.S. citizen children in immigrant families in a state where there were no restrictive laws passed, both with family income of 200% of the federal poverty level or below.

Eligibility for Medicaid/CHIP is presumptive based on family income. Medicaid and CHIP are combined because of the multiplicity of ways that public insurance for children is managed in each state; some states have expanded Medicaid, some have combined programs and/or one-stop application processes, and some have separate CHIP programs. For all states included in the analysis in 2000, the eligibility for children into CHIP which has the most expansive income eligibility of the two programs, was 200% of the FPL or below except for Illinois and South Carolina where income eligibility was 185% or below of FPL. Illinois raised the eligibility threshold to 200% of FPL or below in 2003 and South Carolina raised it to 200% of FPL in 2008.

The data thus defined were pooled for all years (2000–2008) in order to study the changes in enrollment over time. The child is used as the unit of analysis as there may be families where one or more of the children do not qualify for public insurance because of the child’s immigration status or age, while other children in the family may qualify. The outcome measure reflects the likelihood that a child in a family within the income criteria has enrolled in public insurance.

**Fig. 1:**
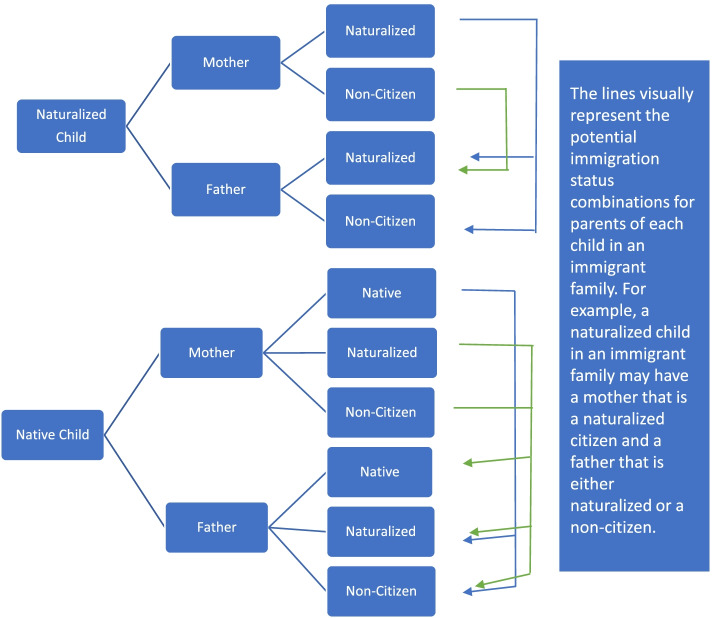
Immigrant families configurations

The foreign-born population includes naturalized US citizens, non-U.S. citizens here legally, and non-U.S. citizens here undocumented. The children in the sample can potentially fall under the following classifications: 1) native child with both parents native (native family, control group) or 2) native/naturalized child with one or more immigrant parents (immigrant family, treatment group). For a breakdown of the possible citizenship and immigrant status configurations of an immigrant family please see Fig. 1.

The Current Population Survey has a non-random, complex survey design so sampling weights are used to produce estimates that would be nationally representative**.** The State Health Access Data Assistance Center (SHADAC) hosts the CPS dataset and created a weight for use with summary health insurance variables (the outcome of interest) in the CPS (HINSWT) that corrects for imputation bias in the March supplement [32]. In addition, the standard errors have been obtained accounting for clustering of children at the state level. As noted, the DD estimator nets out the effect of any unobserved and time-invariant differences between states that had restrictive laws and those that did not in any one year, and together with the other controls for the endogeneity of legislation, allows for a causal interpretation of the results.

### Regression analysis

Using children as the units of observation, I fit linear probability models and obtain difference-in-differences (DD) estimates of the impact of restrictive state laws on enrollment in safety net health insurance. Model One is a linear probability model used to assess the impact of state’s adoption of restrictive laws on children’s enrollment in Medicaid/CHIP.

$${Y}_{cst}={\beta }_{0}+ {\beta }_{1}{X}_{cst}+ {\beta }_{2}{Z}_{st}+{\beta }_{3}ResLa{w}_{st}+ {\beta }_{4}ImmigrantFamil{y}_{cst}+ {\beta }_{5}ResLa{w}_{st}\times ImmigrantFamil{y}_{cst}+ {\beta }_{6}Stat{e}_{ct}+ {\beta }_{7}Yea{r}_{t}+ {\epsilon }_{cst}$$

### Dependent variable

In Model One the response variable has a value of one if child *c* in state *s* at year *t* enrolls in Medicaid or CHIP and is zero otherwise. This is a constructed variable from SHADAC and it indicates whether respondents were covered by Medicaid, SCHIP, or some other non-Medicare, non-military public insurance program [32].

### Independent variables

The model is adjusted for the individual characteristics of the mother and state-specific time-varying characteristics. Attributes of the mother are the mother’s race, ethnicity, citizenship, marital status, number of children, education, and the family’s income in relation to the federal poverty line. The mother’s attributes are considered for two reasons. Research on children’s health insurance enrolment consistently links this enrollment to the mother’s demographic characteristics [13, 33, 34]. In addition, mother’s demographic data is consistently available in this dataset. The model includes any restrictive laws passed in state*s* at any point during year t.

There are family level factors that affect enrollment. Immigrant status and citizenship of parents, as well as ethnicity has been found to be significantly associated with enrollment in public insurance programs among children [35–37]. In one California study the lowest enrollment was not in families with undocumented parents but actually for children of parents with permanent legal residency or “green-card” status [35]. Because parents’ citizenship and ethnicity have been shown in multiple studies to have an effect on enrollment in insurance, these factors will be included in the analysis. Education and family characteristics such as marital status and family size have been linked in previous research to health insurance access [35, 36, 38, 39], while family income is linked to Medicaid eligibility.

The unit of analysis in this study is the child targeted for enrollment in Medicaid/CHIP. A child in an immigrant family takes on a value of 1 if either the mother or father of the U.S. citizen child is non-native.

### State characteristics variables

Since state laws reflect a response to economic and political considerations within a state, it is essential to control for factors that lead to adoption of the laws under consideration in order to address this policy endogeneity. Policy endogeneity is the idea that unobserved factors embedded in the model’s error term could be correlated with the presence of restrictive legislation and with the outcome of interest. Failure to account for omitted and unobserved factors that may be correlated with the adoption of restrictive legislation and outcomes of interest can yield biased impact estimates. These state characteristics are: percent of state residents that are immigrants, the percent of state residents that are not citizens, the percent of state residents over the age of 21 with a high school degree or above,, state net revenue, state unemployment rate, a constructed variable looking at political party concordance of the governing bodies, and previous state generosity with social service benefits to immigrants just prior to the analysis years.

Previous studies have shown that the enactment of state legislation will respond to state level economic, demographic and political variables [40–42]. The variables discussed above represent number of immigrants in the state, the percent of immigrants in the state who cannot vote, state education levels, state economic health, and the political party in power. It is necessary to correct for state time-varying economic conditions that might influence the economic situations of potential Medicaid recipients, and because state economic conditions can affect the resources that can be used to administer the program and support its financing. The state unemployment rate and state-specific fixed effects account for these differences in economic conditions across states. Research shows that party affiliation can have a significant effect on policy outcomes [40, 43].

Previous state generosity in providing social safety net programs to immigrants could affect the likelihood of passage of new anti-immigrant legislation which in turn affect the likelihood of Medicaid take up. The restrictive laws enacted during the 2000–2008 time period under consideration may have been adopted on top of the restrictive laws that already existed at the state level prior to the beginning period of the study. Or potentially, restrictive laws may not have been adopted in the time period under analysis because they were already adopted in the prior time period. States may have for example passed multiple restrictive laws based on immigration status prior to the analysis period and therefore have a more restrictive environment for immigrants without it being captured in the number of restrictive laws being passed during the analysis period. In order to control for potential policy endogeneity, a variable was constructed based on state generosity toward immigrants in 1998, in the wake of welfare reform. The constructed variable is modified from an Urban Institute analysis based on the presence of state funded programs for immigrants to substitute for Medicaid, TANAF, SSI, and food assistance during the five year ban period as well as cost sharing and restrictions on these services [44]. The constructed immigrant generosity variable ranks states from 1–4 respectively as least available, less available, somewhat available, and most available for social safety net services for immigrants prior to the analysis period.

### Interaction term

The estimated coefficient on the state law × immigrant family interaction term is the difference-in-differences estimator which tests whether citizen children in non-native families in states which passed restrictive laws are less likely to enroll in Medicaid/CHIP compared to a control group. The control group consists of children in native families in states that did not pass laws, children in native families in states that passed restrictive laws and children in non-native families in states that did not pass laws. This can answer the question of whether U.S citizen children in non-native families in states that passed restrictive laws were less likely to enroll in Medicaid/CHIP compared to the control groups noted above. A negative estimated coefficient would provide support for the chilling effect.

### Year and state fixed effects

A vector of year-specific dummy variables was included to capture the influence of time-specific change on the outcome on Medicaid/CHIP enrollment. State-specific fixed effects are also included in the model to control for time-invariant differences across all states. The model with state fixed effect was chosen in order to control for average differences across states in any unobservable predictors and reduce the threat of omitted variable bias.

### Weights and clustering

Sample weights were applied to account for any oversampling of population groups in the CPS sampling frame and make the cases reflect the population totals, in order to ensure that the estimated effect truly reflects the behavior of the population under consideration. Because of the potential for intra-group error correlation among observations in the same state, the standard errors were clustered at the state level. Estimation methods need to account for the clustering of observations within states, or the statistical significance of state-level coefficients (our outcome of interest) may be overestimated. Because of the possibility of multiple children in one family being in the analysis, as a sensitivity test the models were also run clustered at the family level and substantive conclusions were unaltered.

### Additional analysis

The analysis was also run for any restrictive laws passed in state *s* at any point during year *t* for laws categorized as education, regulation, and as social welfare in order to determine if certain categories of laws had a significant effect on social welfare use. Education refers to laws that restrict immigrants from receiving funding for secondary education, and also restrict undocumented immigrant access to the public education system. Regulation refers to laws that authorize and/or require law enforcement, government workers, and private citizens to screen individuals for legal status. It also includes limitations on access to identification such as driver’s licenses and requiring local law enforcement to check for legal status during routine traffic stops and other similar events. In addition, this category includes restriction on employment categories, such as requiring the employee to be a U.S. citizen. Social Welfare refers to state measures that further restricts access to means-tested programs from federal regulations. This analysis can be found in Supplement 2.

An immigrant family only sub-sample (Model Two) analysis serves as a sensitivity test for potential dynamic treatment effects of restrictive laws as discussed above. Since citizenship would be a key factor interacting with these laws, the analysis was also run looking at the mother’s citizenship and interaction with restrictive state laws. In this analysis, the control group consists of children in states not passing restrictive laws in that year and having a non-citizen or naturalized parent.

$${Y}_{cst}={\upgamma }_{0}+{\upgamma }_{1}{X}_{cst}+{\upgamma }_{2}{Z}_{st}+{\upgamma }_{3}ResLa{w}_{st}+{\upgamma }_{4}NatMo{m}_{cst}+{\upgamma }_{5}NonCitMo{m}_{cst}+{\upgamma }_{6}ResLa{w}_{st}\times NatMo{m}_{cst}+{\upgamma }_{7}ResLa{w}_{st}\times NonCitMo{m}_{cst}+{\upgamma }_{8}Stat{e}_{ct}+{\upgamma }_{9}Yea{r}_{t}+{\upepsilon }_{cst}$$

One estimation challenge is that this analysis focuses on binary outcomes (enrolled in Medicaid/CHIP or not enrolled) and nonlinear models such as logit or probit are generally best fitted to analyze such outcomes. However, linear probability models have been used successfully to analyze binary outcomes in the context of DD estimation frameworks. For example, many studies looking at insurance market reform use linear probability models to study the effect a set of policy differentials across states since they provide coefficients that show direct estimates of marginal effects [42, 45, 46]. In a nonlinear model the coefficient of the DD model’s interaction term cannot be interpreted as a marginal effect and the sign, and magnitude of the coefficient of the interaction term are not accurate, nor is the standard error for these effects [47]. For this study, the marginal effect of restrictive state laws and their standard errors were obtained directly from linear probability models, and the marginal effect was compared to those derived from logit models and found to be the same.

### Sensitivity tests

Two states among the 20 chosen are potentially influential outliers. Arizona is an outlier due to the large number of restrictive laws that were passed, and Massachusetts is as well, both because it has the least number of restrictive laws of the 20 states and because of Massachusetts’ health reform passed in 2006. As stated previously, the states in this analysis were chosen based on both immigrant growth and total population of immigrants, in order to identify those states most likely to be active in adopting and implementing legislation that might constrain immigrant access to social services. Additionally, a requirement for the analysis is the necessity to have states with an adequate sample size of immigrant families. Since the states that are included with small numbers of laws may lead to an underestimate of the effect of these laws, the state with the smallest number of laws, Massachusetts, was excluded from the analysis in order to check the sensitivity of the findings. Since Massachusetts was unique among all the states in terms of its laws for health insurance during this time period, this exclusion will also address the sensitivity of the finding to the health insurance mandate. Similarly, the analysis was also run excluding the state with the highest number of laws, which was Arizona. These analyses did not show Massachusetts and Arizona to be significant drivers of outcomes, so both states were retained in the final analysis.

In order to understand whether there was an effect based on the number of laws passed in each state in a year, Model One was run substituting any law passed with the number of laws passed in state *s* in year *t*. The interaction term was not included in the model with the quadratic dependent variable. The number of restrictive laws was examined as both total number of laws passed in any given year and as a quadratic expression to determine the marginal effect of an additional law in each year and was found to be non-significant. The tests supported the current model as used in this analysis.

Other sensitivity tests included looking at a sub-sample that includes only low-income children in immigrant families (one or both parents foreign born). In addition, the models were run with single-parent families vs. two-parent families, and looking at the models for families with one child vs. families with multiple children. The results of family composition sensitivity tests can be found in Supplement 2.

### Regression results

Results presented in Table 1 indicate that restrictive laws have a small but statistically significant negative effect (1.8 percentage points less likely to enroll) on enrollment of U.S. citizen children in immigrant families into Medicaid and CHIP (Model One) compared to a control group not likely to be subject to the restrictive legislation. Looking at subsamples of children with and without siblings (Supplement 2), children with siblings in immigrant families demonstrate a chilling effect of 2.1 percentage points (less likely to enroll than children with siblings in the control group). Children in immigrant married families are 2.9 percentage points less likely to enroll than children in native married families. This effect reflects the difference between the treatment and control groups in states where a restrictive law was passed. This effect becomes even more pronounced when looking at just social welfare related legislation (5.5 percentage points less likely to enroll if a child is in the control group). This stronger effect appears to be diluted when looking at all restrictive laws and likely is the driver behind the chilling effect (See Table 2).

**Table 1:** Effect of Restrictive State Laws on U.S. Citizen Children in Low Income Families Use of Medicaid/CHIP: 2000–2008

20 State Analysis	All Children	Children in Immigrant Families
Restrictive Law	− 0.004 (.0076)	-0.003 (.0143)
Restrictive Law*Immigrant Family	− 0.018* (.0097)	NA
Immigrant Family	0.030** (.0136)	NA
Restrictive Law*Non-Citizen Mother	NA	-0.023** (.0108)
Restrictive Law*Naturalized Mother	NA	-0.017 (.0111)

^*^p ≤ .1, **p ≤ .05, ***p ≤ .01

All children includes all children 18 and under in state x at year y with families income 200% or below of federal poverty level. Includes state and year fixed effects. In this all children linear probability regression, data was weighted and the standard error was clustered at the state level. Regression controlled for: mother’s citizenship, race, ethnicity, number of children, and education; family poverty level; State characteristics including, Unemployment rate, % of State Pop. Immigrants, % of State Non-Citizen Immigrants, % of State HS Grad. and above, State Net Revenue, State Gov. Party Concordance and Pre-Analysis State Generosity. Immigrant family linear probability regression also controlled for mother’s citizenship

**Table 2:** Comparing Outcomes for Models One and Two: Looking at Predictors of Medicaid/CHIP Enrollment and Interaction Effects of Restrictive State Laws

20 State Analysis	All Children	Immigrant Families	Only Social Welfare Law (All Children)	Only Social Welfare Law (Immigrant Families)
Restrictive Law	-0.007 (.0074)	0.003 (.0143)	0.011 (.0095)	-0.021 (.0302)
Law*Immigrant Family	-0.018* (.0097)	NA	− 0.055*** (.0140)	NA
Law*Non-Citizen Mother	NA	-0.023** (.0108)	NA	-0.021 (.0350)
Law*Naturalized Mother	NA	-0.017 (.0111)	NA	-0.035 (.0211)
Immigrant Family	0.030** (.0136)	NA	0.031** (.0130)	NA
Citizenship of Mother				
Naturalized	-0.095*** (.0168)	-0.092*** (.0114)	-0.096*** (.0165)	-0.095*** (.0136)
Not a Citizen	-0.007 (.0158)	0.007 (.0150)	-0.008 ( .0157)	-0.000 (.0170)
Poverty Level				
101–150% of FPL	-0.152*** (.0106)	-0.142*** (.0139)	-0.153*** (.0108)	-0.143*** (.0139)
151–200% of FPL	-0.303*** (.0124)	-0.281*** (.0155)	-0.304*** (.0125)	-0.281*** (.0155)
Hispanic Mother				
Hispanic	0.048*** (.0121)	0.063*** (.0185)	0.048*** (.0122)	0.064*** (.0184)
Puerto Rican	0.071*** (.0187)	0.095*** (.0300)	0.071*** (.0187)	0.095*** (.0303)
Unknown	0.032 (.0659)	0.230*** (.0659)	0.032 (.0667)	0.227*** (.0660)
Marital status of Mother				
Married-Spouse Absent	0.013 (.0190)	-0.031 (.0465)	0.013 (.0188)	-0.031 (.0466)
Not Married	0.112*** (.0097)	0.077*** (.0130)	0.112*** (.0098)	0.077*** (.0129)
Education of Mother				
High school Grad	-0.068*** (.0107)	-0.028 (.0181)	-0.068*** (.0107)	-0.028 (.0182)
Some College	-0.100*** (.0121)	-0.054* (.0267)	-0.100*** (.0120)	-0.054* (.0267)
College Graduate	-0.233 (.0125)	-0.093*** (.0290)	-0.233*** (.0125)	-0.093*** (.0290)
Unknown	-0.212** (.0789)	-0.426*** (.0249)	-0.211** (.0782)	-0.425** (.0230)
Race of Mother				
Black	0.055*** (.0103)	0.013 (.0174)	0.055*** (.0102)	0.013 (.0171)
American Indian	0.042 (.0296)	0.063** (.0276)	0.043 (.0296)	0.064** (.0272)
Asian	0.046** (.0172)	0.030** (.0137)	0.045** (.0171)	0.031** (.0138)
Other	-0.418*** (.0177)	-0.456*** (.0145)	-0.420*** (.0170)	-0.456*** (.0120)
Number of Children (Mother)	0.008*** (.0016)	0.007*** (.0020)	0.008*** (.0016)	0.007*** (.0020)
State Unemployment Rate	0.006 (.0055)	-0.002 (.0142)	0.004 (.0055)	-0.008 (.0123)
% of State Pop. Immigrants	0.004 (.0083)	0.014 (.0175)	-.000 (.0078)	0.011 (.0155)
% of State Non-Citizen Immigrants	0.000 (.0014)	0.001 (.0042)	0.001 (.0014)	-0.001 (.0044)
% of State HS Grad. and above	-0.002 (.0060)	-0.011 (.0126)	-0.006 (.0061)	-0.020 (.0122)
State Net Revenue	3.210 (1.3100)	1.130 (1.6600)	-4.720 (1.3800)	4.370 (2.2200)
Governing Party Concordance (D)				
All Republican	0.038** (.0167)	0.051 (.0359)	0.037** (.0152)	0.057 (.0347)
Mixed	0.005 (.0075)	0.008 (.0151)	0.003 (.0075)	0.007 (.0142)
Pre-Analysis State Generosity				
Less Available	0.209* (.1050)	0.293 (.2060)	0.169* (.0973)	0.071** (.0275)
Somewhat Available	0.162*** (.0565)	0.210 (.1486)	0.199*** (.0588)	0.024 (.3170)
Most Available	0.204*** (.0418)	0.317*** (.0839)	0.206*** (.0404)	0.118 (.1726)

^*^p ≤ .1, **p ≤ .05, ***p ≤ .01

All children includes all children 18 and under in state x at year y with family income 200% or below of federal poverty level. Immigrant families includes all children in an immigrant family (at least one non-native parent) 18 and under in state x at year y with family income 200% or below of federal poverty level. Only Social Welfare law includes all children in the sample and also looks at the immigrant subsample, but just includes state measures that grant additional access to means-tested programs or further restricts access to means-tested programs. This was included since it was the only law subset that proved to be significant in previous models. Includes state and year fixed effects. In this linear probability regression, data was weighted and the standard error was clustered at the state level

The interaction term in Model Two looks at mother’s citizenship status (Table 1) in a sub-sample of only immigrant families (one or both parents foreign born) and shows that there is a 2.3 percentage point decrease in the probability of a U.S. citizen child with a non-citizen mother to be enrolled in Medicaid or CHIP in a year in which any restrictive legislation was passed, compared to children with native mothers in immigrant families. This effect is stronger in children without siblings in immigrant families (10.6 percentage points less likely) and among children in non-married immigrant families (4.8 percentage point reduction in enrollment). Please see Supplement 2 for the data table. Unlike when looking at the difference between immigrant and native families, within the immigrant sub-sample, restrictive social welfare legislation does not appear to be driving this chilling effect among children with non-citizen mothers, which is only significant when looking at overall restrictive legislation.

Looking at socio-demographic barriers and facilitators to enrollment (Table 2) independent of the interaction effect, the research results indicate that a higher level of family income is negatively associated with the likelihood of children residing in these families to be enrolled in Medicaid or CHIP. Children living in families that were 101 -150% of the FPL were 15.2 percentage points less likely than families at or below the FPL to be enrolled, and children in families that were 151–200% of the FPL were 30.3 percentage points less likely to be enrolled. Children with mothers who were naturalized citizens were 9.5 percentage points less likely to be enrolled than children with citizen mothers. Children whose mother was not married were 11 percentage points more likely to be enrolled in Medicaid or CHIP than children whose mothers were married. Children whose mother was Black or Asian were more likely to be enrolled than children whose mother was white (5.5 percentage points and 4.6 percentage points respectively). With each additional child, there is a 0.8 percentage point increase in enrollment.

Almost all state characteristics proved to be statistically nonsignificant in predicting whether a child was enrolled. The only exception was the previous state generosity variable, which reveals that a child in a state where social services were most available to immigrants in the period prior to this analysis were 20.4 percentage points more likely to enroll in Medicaid or CHIP compared to states that were classified as having social services least available to immigrants. This is probably indicative of the fact that states that were generous with social services to immigrants are generous overall when it comes to social services. As would be expected, the magnitude of the effect of previous state generosity toward immigrants increased when looking at the immigrant only subsample, going from 20 percentage points more likely for child to enroll in most generous state (previous state generosity variable) in the full sample to 32 percentage points more likely to enroll in the immigrant subsample.

## Discussion

### General characteristics that affect enrollment

There are a number of factors that appear to play an independent role in whether an eligible child is enrolled in Medicaid or CHIP. Family poverty level is strongly associated with a child’s enrollment, with poverty at 100% or below associated with increased likelihood of enrollment, despite the fact that general eligibility requirements are 200% of the federal poverty level or less. This may be due to multiple factors. There may be greater confusion around eligibility requirements among the near poor, from 100 to 200% of federal poverty level, because of additional categorical eligibility restrictions than among families that are 100% of the federal poverty level or below. In addition, families that meet FPL guidelines would fall under Medicaid, which has low cost sharing, while children in families that are above the FPL, especially if they are over the age of 5, would be more likely to qualify for CHIP which tends to have a higher cost sharing that some families may be unable to afford. States use cost sharing with near-poor families’ in public programs in order to address the perceived problem of crowd-out. Multiple studies demonstrate an increase in uninsured among low-income children as cost sharing rises [48–50]. Lastly, higher family income may mean that one of more parents are full time employees and could have access to employer-sponsored insurance. This is also reflected in the differences in children’s enrollment among married and non-married mothers. Children with mothers who were not married were more likely to be enrolled, reflecting both lower incomes among those families and lower chances of having a full-time employed parent with access to employer-sponsored insurance.

In terms of the mother’s demographics, this analysis shows that children with Hispanic mothers were more likely to be enrolled in Medicaid or CHIP (an increase of 4.8 percentage points in full sample, 6.3 percentage points in immigrant sub-sample) than children with non-Hispanic mothers. Children with Black mothers (5.5 percentage points) and Asian mothers (4.6 percentage points) were more likely to be insured by Medicaid or CHIP than children with white mothers in the full sample analysis. This is consistent with the increase in insurance coverage among low-income Hispanic children seen from 1999 to 2002. In this period, the decline in uninsured children was greater for Hispanic and Black children compared to white children due to an increase in coverage in the Hispanic and Black population through Medicaid and CHIP [51]. However, in the immigrant family subsample the significance of race is moderated as a factor in children’s coverage with American Indian (6.3 percentage points) and Asian (3 percentage points) mothers being more likely to enroll their children than white mothers. This may be because the negative effect on likelihood of being insured due to being in an immigrant family reduces racial/ethnic differences in coverage [22, 52].

State characteristics that are associated with enrollment include party concordance and state generosity. An all-Republican state government is more likely to have children enrolled in Medicaid or CHIP then an all-Democratic state government. This may seem counter-intuitive considering the feelings of Republican politicians about smaller government and restricted social services. However, there is some evidence to suggest that states with low per-person income levels and low median household income levels are more likely to vote Republican [53]. Therefore, the state government party make-up may reflect the overall economics of the state not captured by conventional income measures rather than attitudes toward public safety net insurance. The states that had the most available social services for immigrants prior to this analysis were significantly more likely to have higher Medicaid and CHIP enrollment then those states that had the least available services to immigrants, reflecting overall the generous nature of the state toward safety net services.

### Immigrant families and restrictive state laws

Being in an immigrant family in a state and year without a restrictive law means that there is a small but significantly higher likelihood of being enrolled in Medicaid or CHIP compared to native families. This may be due to the type of jobs that low-income immigrant parents are more likely to have, which are less likely to offer health insurance [54]. Children in married immigrant families experienced the most significant decline in enrollment in states with restrictive legislation (2.9% points), followed by children with siblings (2.1% points) and all children in immigrant families (1.8% points). Children with siblings may have experienced a significant decline because of the higher likelihood, compared to families with only one child, that one or more siblings were not born in the U.S. The data shows that children in married families experience a larger chilling effect, which may reflect the fact that immigrant families are more likely to be married compared to native families in this sample.

Looking at children in a subset of only immigrant families (Table 2), the importance of the mothers’ citizenship status on the child’s enrollment becomes clear. Having a non-citizen mother and living in a state that passed a restrictive law significantly reduces the likelihood of a child’s enrollment in public insurance by 2.3 percentage points, which goes up to 4.8 percentage points for children in non-married families and 10.6 percentage points for only children with non-citizen mothers. The effect of having a non-citizen mother in a restrictive law state is ameliorated by having siblings and by living in a married family, both of which would increase the chances that an additional citizen was present in the family. This speaks to the way that a mother’s citizenship interacts with state regulation. Obtaining citizenship is a complex process reflecting multiple factors that include immigration status (need to be a legal permanent resident for at least 5 years), the cost of the process, and English language fluency (including speaking, reading, and writing). The non-citizen category of mothers could also include undocumented immigrants who have the most to lose when accessing social services. A case in point are the arrests of immigrants in “safe spaces” such as schools, churches, courts, and even hospitals for immigration violations [55, 56]. These incidents may make undocumented mothers afraid to access services for their eligible children, especially in states with restrictive legislation aimed at immigrants.

Based on the reduction in coverage among immigrant families in states that passed a restrictive law in that year, we can estimate the number of children that had Medicaid/CHIP coverage in a state without restrictive legislation that would not be covered if the state had adopted restrictive legislation. Using the percentage point reduction as a numerator and the percent of children in immigrant families covered by Medicaid/CHIP in non-restrictive law states as the denominator there would be a 3.5% reduction in children in immigrant families that would have government sponsored health insurance coverage. Using the weighted sample, this is equal to 5.6 million fewer US citizen children covered within these 20 states alone.

### Types of laws

The idea of breaking down restrictive laws by category is to see if there was any independent effect of different types of laws on enrollment is to be able to pinpoint whether laws not directly related to social welfare may have a still have a chilling effect on public insurance access. Looking at the interaction of restrictive laws and immigrant families (Table 2) it is clear that there is a strong interaction between restrictive social welfare laws being adopted and being in an immigrant family on children’s access to Medicaid/CHIP. This strong negative interaction effect appears to be diluted by including regulation and education laws (neither of which had a significant interaction effect on their own). Therefore, the true interaction effect on immigrant families seems to be driven by restrictive social welfare laws. There would be a 10.6% reduction in children in immigrant families that would have government sponsored health insurance coverage in states that passed restrictive social welfare laws. Using the weighted sample 17 million fewer children would have Medicaid /CHIP coverage if all states had adopted restrictive social service welfare laws. Looking at U.S. citizen children in immigrant families compared to the control group, it seems that the difference in Medicaid/CHIP enrollment may be attributed to laws that specifically target social welfare access for immigrants.

Looking at the interaction between mother’s citizenship and restrictive laws it is clear that the opposite is true. Here we can see a true chilling effect of having restrictive legislation aimed in general at immigrants. Within the immigrant family subsample, the impact of having a non-citizen mother in a state with restrictive laws is a 2.3 percentage point reduction in enrollment for U.S. citizen children. This is despite non-significant results in each of the three categories of laws alone, showing that an overall negative legislative environment in the states towards immigrants (including laws related to job, ID, and welfare program access) creates this chilling effect. All restrictive state laws related to immigrants appear to have an interaction affect with mothers’ citizenship status, which reduces enrollment, while specific social welfare restriction laws affect immigrant families in general. These results suggest that the findings are robust and largely similar to the full sample, validating that the results are not masking dynamic treatment effects.

### Limitations

One important limitation of the data in this study is that it is not possible to determine if the mothers are undocumented immigrants. The non-citizen category includes both legally present non-citizens and those non-citizens who are in the country without documentation. Parents’ willingness to enroll their child in Medicaid/CHIP and their response to restrictive state laws may be different between documented and non-documented non-citizen immigrants.

The period of data analysis was selected to align with a period between the implementation of important relevant federal policy (after PRWORA and before the ACA) in order to isolate the effects of state level policy. An additional analysis that considers the time period following implementation of the ACA, which research suggests may have expanded access to health insurance for children in immigrant families, will be an important next step.

Correctly reporting public insurance is a problem across multiple surveys. While uninsured estimates are relatively accurate across all surveys, the CPS does have problems with coverage misclassification, likely due to the yearlong recall period [57]. One study comparing the CPS to the Medicaid Statistical Information System (MSIS) found that composite Medicaid/CHIP reporting (as used in this analysis) had a smaller reporting error than CHIP alone [58].

Enforcement of these laws is not possible to determine, and rigorous enforcement may create a greater chilling effect [59]. Another limitation, which may be remedied through further studies, is the inability of this study to determine the cause of the chilling effect. It may be that media coverage of the restrictive legislation is the driver behind this effect, or it may be a generally anti-immigrant climate in the state.

## Conclusion

The key message from this study is that there is a chilling effect among immigrant families, which decreases their children’s likelihood of enrollment in Medicaid/CHIP related to the passage of restrictive legislation based on immigration status. As states enact restrictive legislation related to immigration status, this could create a widening gap in health insurance coverage for eligible children who live in immigrant families compared to children in native families. National Conference of State Legislatures Report showed that in 2019 there were 181 laws related to immigration and immigrants passed across 49 states and Puerto Rico [60]. The chilling effect due to restrictive state laws is an ongoing issue that creates unintended consequences for U.S. citizen children in immigrant families’ access to public insurance.

This research is one of the first to specifically designed to estimate the magnitude of a chilling effect of restrictive state laws. Previously a chilling effect was mostly inferred from an overall drop in Medicaid enrollment after passage of federal laws, or enrollment disparities between Medicaid eligible citizen children with immigrant and non-immigrant parents [22, 23]. The results from this analysis show a specific magnitude of effect of these laws and demonstrates that this chilling effect is not due to other potential factors like a change in state economic outlook, but instead directly attributable to these restrictive laws. The strength and importance of this finding is that we can quantify the harm dome to children living in states with restrictive laws. This study also allows shows an important distinction between Medicaid/CHIP, which is a benefit that accrues to one individual, and previous research on food stamp/SNAP benefits which are a family-level benefit, when it comes to the chilling effect of restrictive laws [28]. In addition, the research indicates that laws aimed specifically at restricting social service access create this chilling effect for immigrant families, while laws aimed at education, job restriction, and social welfare have a significant impact on access to pubic insurance for a particular subgroup- those children with non-citizen mothers.

The unintended consequence of restrictive state legislation aimed at immigrants is the reduction in access to Medicaid and CHIP by low-income U.S. citizen children living in immigrant families. This in turn decreases the chances that that these children will have well-child visits, increases the likelihood of emergency room visits, and increases unmet medical needs [61, 62]. The results of this study strongly support the idea of a chilling effect and suggest the need for outreach and education to foreign-born parents in order to ensure that all eligible children are enrolled in Medicaid/CHIP.

Outreach programs need to be aware of the barriers to enrollment for eligible children in immigrant families created by restrictive state legislation, as well as the importance of the citizenship of the mothers in their willingness to sign-up their children for Medicaid/CHIP. It is important to acknowledge that the chilling effect stems from social welfare specific legislation, but expands beyond the targeted population and negatively impacts children who should have access to these programs. The population that was the most vulnerable to the chilling effect were low-income citizen children with non-citizen mothers, whose enrollment in Medicaid/CHIP was adversely affected by any state-level restrictive immigrant related legislation.

## Supplementary Information

**Additional file 1.** 

**Additional file 2.** 

## Data Availability

The Current Population Survey March Supplement database analyzed during the current study are available online from: https://cps.ipums.org/cps/index.shtml [25]. The state law data generated during this project is available from Figshare (https://doi.org/10.6084/m9.figshare.7960967.v3) [27].
