# Supplementary material for: Do state laws reduce uptake of Medicaid/CHIP by U.S. citizen children in immigrant families: evaluating evidence for a chilling effect
Source: Int J Equity Health. 2022 Apr 12;21:50. doi: 10.1186/s12939-022-01651-2 (PMC9006602; doi:10.1186/s12939-022-01651-2)
Supplement: Supplementary file 1 — Additional file 1. [file 12939_2022_1651_MOESM1_ESM.docx]

**Supplement One: State Law Dataset**

**Methodology of Law Identification**

The dataset is restricted to states in the continental United States that (1) in 2000 and 2008 were at or above the U.S. average for percentage of foreign-born population (13 total), and (2) states that ranked in the top 10 percent in terms of change in foreign-born population from 2000 to 2008. This criterion was applied to identify states that had reason to enact legislation due to a large population of immigrants and to also consider states with significant growth in their immigrant populations over time. This population change could lead to legislation to address growing concerns in the state regarding immigrants in the community. Twenty states were included in the analysis based on these criteria. States representing criteria one include: Arizona, California, Connecticut, Florida, Illinois, Massachusetts, New Jersey, New York, Rhode Island and Texas. Sates representing criteria two include: Alabama, Arkansas, Delaware, Georgia, Kentucky, Mississippi, North Carolina, South Carolina and Tennessee. Nevada meets both criteria for inclusion. Table 1 below shows the number of laws for each state over the target time period that met the inclusion criteria.

*Identification and categorization of state laws*

To identify the applicable state laws, a search was conducted through the Lexis Nexus Federal and State Codes, Advanced Legislative Service-50 states, DC, PR, and VI combined. The terms used,

alien OR immigra! OR "nonimmigra!" or citizenship OR noncitizen OR "non-citizen" OR "not a citizen" OR undocumented OR "lawful presence" OR "legal! presen!" OR "legal permanent residen!" OR "lawful permanent resident" OR migrant OR "employment eligibility" OR "unauthorized worker" OR "human trafficking" OR refugee AND date > 31 December 1999 AND date <01 January 2009,

were based on legislative searches conducted by the Migration Policy Institute [1].

*Inclusion and Exclusion Criteria*

The first part of the legislative analysis eliminated legislation from the search that did not involve any active change dealing with immigrants in the year that the bill was passed. Many times bills will be approved in order to affect small changes to the wording or add clarification that does not in any way impact on immigrants. These bills were excluded from the analysis as they did not reflect any active changes to the state law affecting the population of interest. In addition, each bill was checked to make sure that it was passed by the governor and in states with line item vetoes that the part of the legislation dealing with immigrants was not vetoed.

For this dataset a number of laws were excluded from consideration. The legislation that was excluded includes program funding bills, laws for the purpose of celebration or commemoration, laws that regulate alien business or taxes, development of taskforces or studies, legislation related to divestment, laws regarding migrant housing and education, laws related to child support, adoption, or custody, laws related to selective service, and those laws related to identity theft (except as specific to fraudulent citizenship or visa identification). If two versions of a bill are passed the same year that offer the same language and/or impact on immigrants within the state, for example similar laws passed in the same year in the house and senate, then the law was only counted once.

Then the legislation was checked to make sure that it met inclusion criteria. The year of the legislation is the year that the bill was passed into law. The law had to have been passed within the 2000 through 2008 legislative sessions in one of the twenty states under analysis. The law must either restrict or expand access, rights, or community integration related activities based on immigration status. The remaining legislation was then classified to determine if the law affected education, regulation, or social welfare, and then further to classify the law as restrictive or non-restrictive regarding immigrant rights and access.

*Education* refers to laws that either allow for or restrict immigrants from receiving funding for secondary education, and also restrict or protect undocumented immigrant access to the public education system. This legislation would have a direct impact on the ability of immigrants over time to develop the human capital and credentials necessary to obtain access to well-paying employment and health benefits. *Regulation* refers to laws that authorize and/or require law enforcement, government workers, and private citizens to screen individuals for legal status. An example of this would be an employer who is required to screen their employees to ensure that they are permitted to work in this country. It also includes limitations on access to identification such as driver’s licenses and requiring or prohibiting local law enforcement to check for legal status during routine traffic stops and other similar events. In addition, this category includes restriction on employment categories, such as requiring the employee to be a U.S. citizen. These laws may make it difficult for an immigrant to gain access to jobs either directly or due to employer or immigrant fears, and may restrict access to transportation, banking, etc. These restrictions can have a direct effect on the ability of the immigrant to integrate into the community and provide for themselves and their family. Both categories of these laws may also have a chilling effect, creating a climate of fear whereby restrictive legislation in the areas of education and regulation create an environment where immigrant families will choose not to access social safety net programs. Finally, *Social Welfare* refers to state measures that grant additional access to means-tested programs or further restricts access to means-tested programs from federal regulations. An example of this would be regulations that restore Medicaid eligibility after the federal five-year waiting period and/or provide state sponsored healthcare for immigrants who do not qualify for federal assistance. If there were multiple parts of one bill that fall under these categorizations, such as in an omnibus bill, each part was counted separately if it could have separate effects on the immigrant population in the state.

**Table 1: Laws that fit criteria for selected states in the time period 2000-2008**

| **2000-2008** | **Total laws that meet criteria** | **Large Immigrant population** | **Immigrant population growth over time** |
| --- | --- | --- | --- |
| Alabama | 33 |  | X |
| Arkansas | 12 |  | X |
| Arizona | 42 | X |  |
| California | 32 | X |  |
| Connecticut | 23 | X |  |
| Delaware | 12 |  | X |
| Florida | 33 | X |  |
| Georgia | 18 |  | X |
| Illinois | 24 | X |  |
| Kentucky | 9 |  | X |
| Massachusetts | 5 | X |  |
| Michigan | 30 |  | X |
| Nevada | 26 | X | X |
| New Jersey | 10 | X |  |
| New York | 18 | X |  |
| North Carolina | 12 |  | X |
| Rhode Island | 11 | X |  |
| South Carolina | 24 |  | X |
| Tennessee | 20 |  | X |
| Texas | 20 | X |  |

**Descriptive Statistics: Discussion of Laws by State and Over Time**

*Education Laws*

The education category has the least number of total laws passed in this eight-year period under analysis. There were a total of 23 laws passed over the eight years in the twenty states that fit within this category. Of these 23 laws, 10 expanded the access of immigrants to higher education and 13 restricted access. The majority of laws in this category address in-state tuition requirements and financial aid to colleges. Some laws are related to grants, scholarship, and/or loan forgiveness for specific high-need professions working in underserved areas such as teachers and social workers. Only one law was related to primary education. In Massachusetts, in 2002, a law was passed stating that immigrant students could no longer be taught in bilingual or native language classes and instead had to attend English-only classrooms.

*Social Welfare Laws*

In the period of 2000-2008 there were a total of 97 laws passed in the 20 states that dealt directly with immigrants and access to social services. Out of those 97 laws, 59 (across 15 states) expanded access to the public safety net. These laws expanding access generally restored access to legal immigrants who no longer qualified for federal funds due to the PRWORA legislation. In some cases this restoration was only for specific groups such as pregnant women, the disabled, or the elderly. These expansions in some states included access to all public benefits and in some laws addressed specific access to programs such as Medicaid and TANF. No states gave access to social services for undocumented immigrants except in the case of domestic violence and human trafficking. There were five laws passed that specifically guaranteed access to public services regardless of immigration status to individuals who were victims of domestic violence (CA, 2006; NY, 2008) and human trafficking (NJ, 2005; CA, 2006; NY, 2007; NC, 2007).

The restrictive legislation limits the access of legal immigrants to social services including cash assistance, disability services, and healthcare across 14 different states. As can be seen from these numbers, some states passed both expansive legislation, usually for a specific sub-group of immigrants, and restrictive legislation. For example, California passed one law restricting some access to Medicaid but passed 10 laws related to expansion of services such as food stamps, supplemental security income and healthcare. In one particularly interesting law, California required a notice to go out with applications for free and reduced price school lunches stating specifically that no information on the application would be shared with immigration services, in an attempt to encourage use of this social service among immigrant families. This demonstrates the state’s awareness that fear of immigration consequences can keep children in immigrant families from accessing needed basic services.

*Regulation Laws*

Regulation is the most wide-ranging category of the three types of laws. Its major focuses includes regulating specific job categories to either expand immigrant access for certain skilled jobs in underserved areas such as physicians, dentists, and teachers, or restricting immigrant access to jobs requiring licensing such as contractors, bail bondsman, brokers, and cosmetologists. The mechanism to restrict access in these cases is the application for a license to practice the profession in the state. Additional legislation aimed at employment includes required verification of workers’ immigration or citizenship status. There are 108 laws, across 20 states, which deal directly with regulation of the employment of immigrants. Access to firearms is another type of regulatory law, with 11 laws across eight states, all of which restrict immigrant access.

There are a number of regulations aimed at protection of immigrants against human trafficking and predatory legal services. The human trafficking laws expand the scope of coercive practices to include things such as confiscating immigration documents and/or threating to contact immigration authorities. These laws create criminal penalties for engaging in human trafficking. Some laws also target the international bride trade. There are 21 laws across eleven states regarding consumer protection from fraudulent or predatory immigration legal services by notary publics.

There are regulations that deal with non-citizen in the courts and jail systems, including some designed to ensure that prisoners know how a conviction may affect their ability to stay in the country as well as mechanisms for deporting prisoners and parolees. There has been a lot of media attention to the regulation of IDs such as driver’s licenses for immigrants, and 31 laws regarding IDs were passed over the eight year time span. The majority of these (22) were restrictive.

*Excluded Laws and Resolutions*

Laws and resolutions not included in the analysis may still reflect the climate of the state. Studies and commissions were not included in the analysis because while they may lead to legislative change at a later date, they have no practical impact on the residents of the state. They can however be indicative of the climate of the legislature. Non-binding resolutions can indicate negative feelings toward immigrants’ impact on state’s budget and social services. For example, a 2000 resolution in Kentucky called for the state to investigate the impact of immigration on public services. Another example is the 2008 Alabama resolution calling for the President and Congress to “secure the borders and protect the workforce.” The majority of these non-binding resolutions reflect a distrust of the immigrant population and its impact on the country.

The difficulty of passing legislation in an environment where the governor and the legislature fail to agree can be seen in the power of the line-item veto. For example, in 2004 in Massachusetts the governor vetoed a law allowing in-state tuition for legal immigrants who were in high school for three years in the state. That same year the Massachusetts governor also vetoed provision of social services to disabled legal immigrants who did not qualify under federal law. Massachusetts was the only state included in the analysis where the governor exercised the line-item veto power on laws related to immigrants within the study period. Because the laws were blocked from going into effect they are not reflected in enumeration of the state laws. Nevada, North Carolina, and Rhode Island are the only states out of the 20 included where the governors do not have some form of line-item veto power. Even in these states however the governor can choose to veto the entire piece of legislation.

**References**

1. Laglagaron L, Rodríguez C, Silver A, Thanasombat S. Regulating immigration at the state level: Highlights from the database of 2007 state immigration legislation and the methodology. Migration Policy Institute. 2008 Oct.
