# Supplementary material for: Do state laws reduce uptake of Medicaid/CHIP by U.S. citizen children in immigrant families: evaluating evidence for a chilling effect
Source: Int J Equity Health. 2022 Apr 12;21:50. doi: 10.1186/s12939-022-01651-2 (PMC9006602; doi:10.1186/s12939-022-01651-2)
Supplement: Supplementary file 2 — Additional file 2. [file 12939_2022_1651_MOESM2_ESM.docx]

**Supplement Two: Effects of Types of Laws and Sensitivity Tests**

**Differential Effects of Types of Restrictive State Laws on U.S. Citizen Children in Low Income Families’ Use of Medicaid/CHIP: 2000-2008**

| 20 State Analysis | **All Restrictive Laws** | **Restrictive Education laws** | **Restrictive**  **Regulation Laws** | **Restrictive Social Welfare Laws** |
| --- | --- | --- | --- | --- |
| **All Families Sample** | | | | |
| Restrictive Law | −0.004  (.0076) | 0.028  (.0172) | −0.006  (.0081) | 0.011  (.0095) |
| Law*Immigrant Family | −0.018*  (.0097) | 0.009  (.0301) | -0.011  (.0130) | −0.055***  (.0140) |
| Immigrant family | 0.030**  (.0136) | 0.022  (.0150) | 0.026**  (.0110) | 0.031**  (.0130) |
| **Immigrant Families Sample** | | | | |
| Restrictive Law | -0.003  (.0143) | 0.025  (.0361) | 0.003  (.0237) | −0.021  (.0301) |
| Law*Non-Citizen Mother | -0.023**  (.0108) | 0.010  (.0310) | −0.018  (.0160) | −0.021  (.0350) |
| Law*Naturalized Mother | -0.017  (.0111) | 0.031  (.0228) | −0.021  (.0198) | −0.035  (.0211) |

*p<.1, **p<.05, ***p<.01

Notes: All families includes all children 18 and under in state x at year y with family income 200% or below of federal poverty level. Includes state and year fixed effects. Immigrant families includes all children in an immigrant family (at least one non-native parent) 18 and under in state x at year y with family income 200% or below of federal poverty level.

Education refers to laws that either allow for or restrict immigrants from receiving funding for secondary education, and also restrict or protect undocumented immigrant access to the public education system. Regulation refers to laws that authorize and/or require law enforcement, government workers, and private citizens to screen individuals for legal status. Social Welfare refers to state measures that grant additional access to means-tested programs or further restricts access to means-tested programs from federal regulations.

In this linear probability regression, data were weighted and the standard error was clustered at the state level. Regression controlled for: mother’s citizenship, race, ethnicity, number of children, and education; family poverty level; State characteristics including, Unemployment rate, % of State Pop. Immigrants, % of State Non-Citizen Immigrants, % of State HS Grad. and above, State Net Revenue, State Gov. Party Concordance and Pre-Analysis State Generosity.

**Effect of Restrictive State Laws on U.S. Citizen Children in Low Income Families Use of Medicaid/CHIP: 2000-2008**

| **20 State Analysis** | **All Children** | **Children**  **Without Siblings** | **Children With Siblings** | **Children in Married Families** | **Children in Non-Married Families** |
| --- | --- | --- | --- | --- | --- |
| Restrictive Law | −0.004  (.0076) | −0.017  (.0124) | −0.001  (.0090) | 0.005  (.0121) | -0.013  (.0072) |
| **Restrictive Law*Immigrant Family** | −0.018*  (.0097) | 0.000  (.0168) | −0.021*  (.0102) | -0.029*  (.0157) | 0.009  (.0193) |
| Immigrant Family | 0.030**  (.0136) | 0.027  (.0217) | 0.030**  (.0135) | 0.059***  (.0155) | -0.009  (.0148) |

*p<.1, **p<.05, ***p<.01

Notes: All children includes all children 18 and under in state x at year y with families income 200% or below of federal poverty level. Includes state and year fixed effects. In this linear probability regression, data was weighted and the standard error was clustered at the state level. Regression controlled for: mother’s citizenship, race, ethnicity, number of children, and education; family poverty level; State characteristics including, Unemployment rate, % of State Pop. Immigrants, % of State Non-Citizen Immigrants, % of State HS Grad. and above, State Net Revenue, State Gov. Party Concordance and Pre-Analysis State Generosity.

**Effect of Restrictive State Laws on U.S. Citizen Children in Low Income Immigrant Families’ Use of Medicaid/CHIP: 2000-2008**

| 20 State Analysis | **Children in Immigrant Families** | **Children w/out Siblings in Immigrant Families** | **Children with Siblings in Immigrant Families** | **Children in Married Immigrant Families** | **Children in Non-Married Immigrant Families** |
| --- | --- | --- | --- | --- | --- |
| Restrictive Law | -0.003  (.0143) | 0.072*  (.0357) | −0.006  (.0156) | -0.010  (.0172) | 0.026  (.0274) |
| **Restrictive Law*Non-Citizen Mother** | -0.023**  (.0108) | −0.106**  (.0406) | −0.013  (.0125) | −0.008  (.0196) | −0.048*  (.0260) |
| Restrictive Law*Naturalized Mother | -0.017  (.0111) | −0.092*  (.0475) | −0.006  (.0111) | −0.012  (.0121) | -0.007  (.0359) |

*p<.1, **p<.05, ***p<.01

Notes: Includes state and year fixed effects. In this linear probability regression, data was weighted and the standard error was clustered at the state level. Regression controlled for: mother’s citizenship, race, ethnicity, number of children, and education; family poverty level; State characteristics including, Unemployment rate, % of State Pop. Immigrants, % of State Non-Citizen Immigrants, % of State HS Grad. and above, State Net Revenue, State Gov. Party Concordance and Pre-Analysis State Generosity.
